# *Cratylia mollis* 1, 4 Lectin: A New Biotechnological Tool in IL-6, IL-17A, IL-22, and IL-23 Induction and Generation of Immunological Memory

**DOI:** 10.1155/2013/263968

**Published:** 2013-03-18

**Authors:** Priscilla Stela Santana de Oliveira, Moacyr Jesus Barreto de Melo Rêgo, Rafael Ramos da Silva, Mariana Brayner Cavalcanti, Suely Lins Galdino, Maria Tereza dos Santos Correia, Luana Cassandra Breitenbach Barroso Coelho, Maira Galdino da Rocha Pitta

**Affiliations:** ^1^Laboratório de Imunomodulação e Novas Abordagens Terapêuticas (LINAT), Universidade Federal de Pernambuco (UFPE), Avenida Prof. Moraes Rêgo 1235, Cidade Universitária, 50670-901 Recife, PE, Brazil; ^2^Laboratório de Glicoproteínas, Centro de Ciências Biológicas, Universidade Federal de Pernambuco (UFPE), Avenida Prof. Moraes Rêgo 1235, Cidade Universitária, 50670-901 Recife, PE, Brazil

## Abstract

*Cratylia mollis* lectin has already established cytokine induction in Th1 and Th2 pathways. Thereby, this study aimed to evaluate Cramoll 1, 4 in IL-6, IL-17A, IL-22, and IL-23 induction as well as analyze immunologic memory mechanism by reinducing lymphocyte stimulation. Initially we performed a screening in cultured splenocytes where Cramoll 1, 4 stimulated IL-6 production 5x more than ConA (*P* < 0.05). The same behavior was observed with IL-22 where the increase was greater than 4x. Nevertheless, IL-17A induction was similar for both lectins. In PBMCs, the same splenocytes course was observed for IL-6 and IL-17A. Concerning the stimulation of IL-22 and IL-23 Cramoll 1, 4 was more efficient than ConA in cytokines stimulation mainly in IL-23 (*P* < 0.01). Analyzing reinduced lymphocyte stimulation, IL-17A production was higher (*P* < 0.001) when the first stimulus was realized with Cramoll 1, 4 at 1 **μ**g/mL and the second at 5 **μ**g/mL. IL-22 shows significant differences (*P* < 0.01) at the same condition. Nevertheless, IL-23 revels the best response when the first stimuli was realized with Cramoll1, 4 at 100 ng/mL and the second with 5 **μ**g/mL. We conclude that the Cramoll 1, 4 is able to induce IL-6, IL-17A, IL-22, and IL-23 cytokines *in vitro* better than Concavalin A, besides immunologic memory generation, being a potential biotechnological tool in Th17 pathway studies.

## 1. Introduction

Cramoll lectins are purified from *Cratylia mollis* leguminous seeds; until now four isoforms were discovered on this species, among then the association of forms 1 and 4 (Cramoll 1, 4). Like the well-established lectin from *Canavalia ensiformis*, Concanavalin A (ConA), Cramoll has its carbohydrate recognition site specific for glucose/mannose. Isoforms 1 and 1, 4 have similar characteristics to ConA [1] not only in its structural arrangement but also on its *in vitro *activity as T lymphocyte mitogen [2].

Studies comparing Cramoll 1, 4 and ConA have proved that the high biotechnological potential of Cramoll 1, 4 in relation to ConA such as tumor molecular probes, acting as breast [3], prostate cancer biomarker [4], and *in vitro* induction of cytokines belonging to the classical Th1 immune pathway in mice splenocytes [5, 6]. Furthermore Cramoll 1, 4 also showed healing properties in mouse induced lesions [7], anticancer effects, when encapsulated into liposomes [8], and induced epimastigotes death by necrosis in an *in vitro* tripanosomiasis model [9]. 

On the other hand the exact role of Cramoll and ConA in the induction of cytokines belonging to Th17 pathway has not been reported yet [10]. In this pathway, the synergistic action of IL-6 and transforming growth factor beta (TGF-*β*) on naive T cells, induce differentiation into Th17 lymphocytes through the activation of transcription factor regulator (ROR-*γ*t) becoming essentially producers of IL-17A, IL-17F, and IL-22, with IL-23 being responsible for the phenotype maintaining [11]. The Th17 pathway mediates protective actions in bacterial and fungal infections, but in human autoimmune diseases it has deleterious effects compromising the patient's clinical evolution, becoming a great target on experimental research [12]. Therefore, this study aimed to evaluate the stimulation effectiveness of ConA and Cramoll 1, 4 in IL-6, IL-17A, IL-22, and IL-23 production as well as analyze the mechanism of immunologic memory boosting a secondary response primary immunization by Cramoll 1, 4.

## 2. Materials and Methods

### 2.1. Lectins

Seeds of *C. mollis* Mart. (camaratu bean) were collected in the State of Pernambuco (Brazil), and the lectin (Cramoll 1, 4) was purified according to Correia and Coelho, 1995 [13]. The seed extract (10% w/v in 0.15 M NaCl) was ammonium sulphate fractionated (40–60%) and then purified by affinity chromatography on a Sephadex G-75 column. Cramoll 1, 4 elution was performed with 0.3 M glucose in 0.15 M NaCl. ConA was purchased from Sigma-Aldrich.

### 2.2. Animals

Experimental assays utilized mice BALB/c (male, 45 days old). The animals (*n* = 6) were raised and maintained at Laboratório de Imunopatologia Keizo Asami (LIKA), Universidade Federal de Pernambuco (UFPE), Recife, Brazil. The guidelines of the Animals' Ethical Committee of our institution were followed after their approval.

### 2.3. Preparation of Splenocytes

The spleen of each mouse was removed aseptically and placed in a Petri dish containing RPMI-1640 (Gibco). In a vertical flow, each spleen was transferred to another Petri dish where they were soaked. The cell suspensions obtained from each spleen were filtered in a 40 *μ*m nylon cell strainer (BD bioscience) and then transferred to centrifuge tubes. Spleen homogenates were then centrifuged twice at 300 g for 10 min. Cells were then treated with 1X RBC lysis buffer (eBiosciences). Cells were counted in a Neubauer chamber, and cell viability was determined by the trypan blue exclusion method being used when viability was >98%.

### 2.4. PBMCs Purification

Peripheral blood mononuclear cells (PBMC) were obtained from heparinized blood from healthy, nonsmoking donors who had not taken any drugs for at least 15 days prior to sampling that was collected (*n* = 6), and the PBMC were isolated via a standard method of density-gradient centrifugation over Ficoll-Hypaque (GE Healthcare). Cells were counted in a Neubauer chamber, and cell viability was determined by trypan blue exclusion method. Cells were only used when viability was >98%. All donors signed an informed consent form, and the study was approved by the human research ethics committee of UFPE Health Sciences Center (CEP/CCS/UFPE N0 145/09).

### 2.5. Splenocytes and PBMCs Cultures

Splenocytes and PBMC (1 × 10^6^ cell/well) were cultured in 24-well plates in RPMI-1640 (Gibco) supplemented with 10% fetal bovine serum (Gibco), HEPES 10 mM (Gibco), and penicillin/streptomycin 200 U/mL (Gibco). The cells were stimulated with ConA and Cramoll 1, 4 at 100 ng/mL, 1 *μ*g/mL, and 5 *μ*g/mL or not and incubated at 37°C in a humidified 5% CO_2_ incubator. Unstimulated (USC) cells were used as control.

### 2.6. Cytokine Titration

Cytokines in the supernatants of splenocyte cultures were assayed with ELISA mouse kits according to the manufacturer's instructions. The lower limits of ELISA kits detection were 7.8 pg/mL for IL-6 (BD Biosciences), 3.9 pg/mL for IL-17A (eBiosciences), 7.8 pg/mL from IL-22 (eBiosciences), and 15.6 from IFN*γ* (BD Biosciences). Cytokines were assayed in PBMCs culture from healthy subjects with ELISA human kits according to the manufacturer's instructions. The lower limits of detection for the ELISA human kits were 15.6 pg/mL for IL-6 (BD Biosciences), 3.9 pg/mL for IL-17A (eBiosciences), 15.6 pg/mL for IL-22 (eBiosciences), and 15.62 for IL-23p19 (eBiosciences) levels were measured at 48 h and 24 h after restimulation by human kits.

### 2.7. Statistical Analysis

All experiments were performed at least three independent times before statistical analysis. The test used was one-way ANOVA in which differences were considered significant when *P* < 0.05. In all graphs, bars represent mean value ± standard deviation.

## 3. Results

### 3.1. Cramoll 1, 4 Induces IL-6, IL-17A, and IL-22 Production in BALB/c Splenocytes Cultures

**Figure 1:**
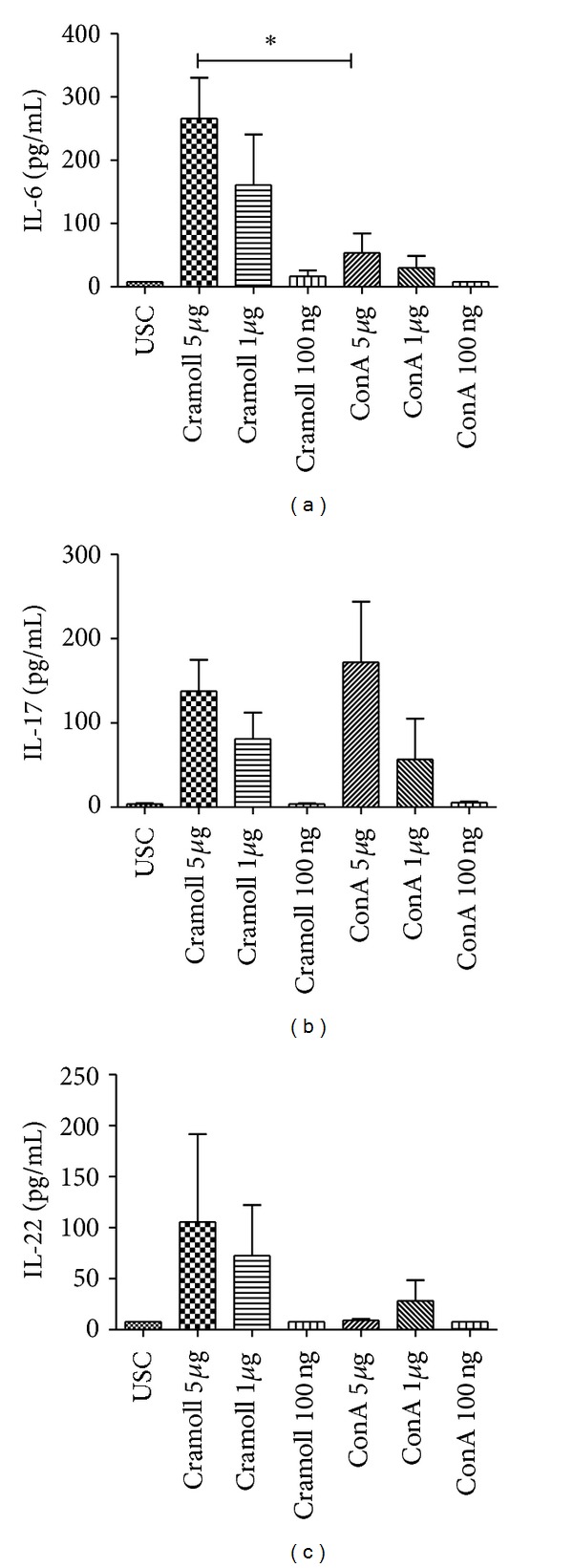
Production of IL-6 (a), IL-17 (b), and IL-22 (c) cytokines (pg/mL) from BALB/c mice splenocyte cultures in relation to different stimuli concentrations of Cramoll 1, 4 and ConA.

Initially, a Cramoll 1, 4 cytotoxicity assay was performed, and no toxicity was observed in all tested concentrations (100 ng/mL–5 *μ*g/mL). These data were previously confirmed by our group [14]. When the ability of Cramoll 1, 4 to induce IL-6, IL-17A, IL-22 cytokine production was evaluated, a significant increased production of IL-6 and IL-22 at all concentrations tested was obtained when compared with ConA (Figure 1). The best dose to stimulate cytokine production was 5 *μ*g/mL where IL-6 production by Cramoll 1, 4 stimulus (279.8 pg/mL) was 5 times higher than ConA (53.4 pg/mL) *P* < 0.05 (Figure 1(a)). The same behavior was observed for IL-22 which production was 117.6 pg/mL when stimulated with Cramoll 1, 4 and 30.5 pg/mL when stimulated with ConA revealing that Cramoll has 3.8 times more induction power of IL-22 production (Figure 1(b)), although the difference was not statistically significant. On the other hand, similar results between Cramoll 1, 4 and ConA were found in IL-17A production (145.4 pg/mL and 117.2 pg/mL), respectively (Figure 1(c)). No IL-17A significative differences were observed to both lectins in assayed concentration.

### 3.2. Cramoll 1, 4 Induces IL-6, IL-17A, IL-22, and IL-23 Production in PBMC Culture

**Figure 2:**
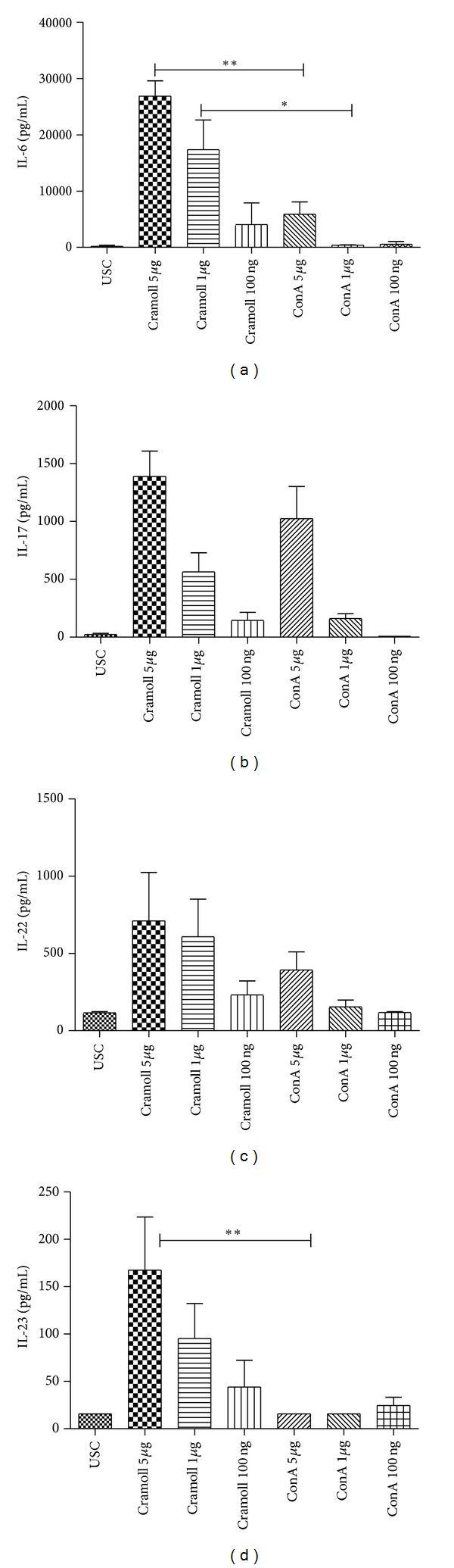
Production of IL-6 (a), IL-17 (b), IL-22 (c), and IL23 (d) in human PBMC culture stimulated with different concentration of Cramoll 1, 4 and ConA.

The confirmation of Th17 pathway cytokine production in murine splenocytes triggered our interest in analyzing the effects of Cramoll 1, 4 and ConA in human PBMCs. Cramoll 1, 4 was effective in stimulating the production of IL-6 better than ConA in all tested concentration being significative at 5 *μ*g (*P* < 0.01) and 1 *μ*g (*P* < 0.05) (Figure 2(a)). For IL-17A (Figure 2(b)) Cramoll 1, 4 was more effective to stimulate cytokine production in all tested concentration, but no result was statistically significative (Figure 2(b)). The stimulation of IL-22 (Figure 2(c)), that with IL-17A form the main cytokines produced by Th17, was more effective with Cramoll 1, 4 than ConA. Cramoll 1, 4 was also more efficient than ConA in IL-23p19 stimulation. This cytokine is responsible for maintenance of Th17 pathway which production by Cramoll 1, 4 on 5 *μ*g/mL was 167 pg/mL and for ConA was 15 pg/mL (*P* < 0.01) showing a high significant difference.

### 3.3. Cramoll 1, 4 Induces Immunological Memory

**Figure 3:**
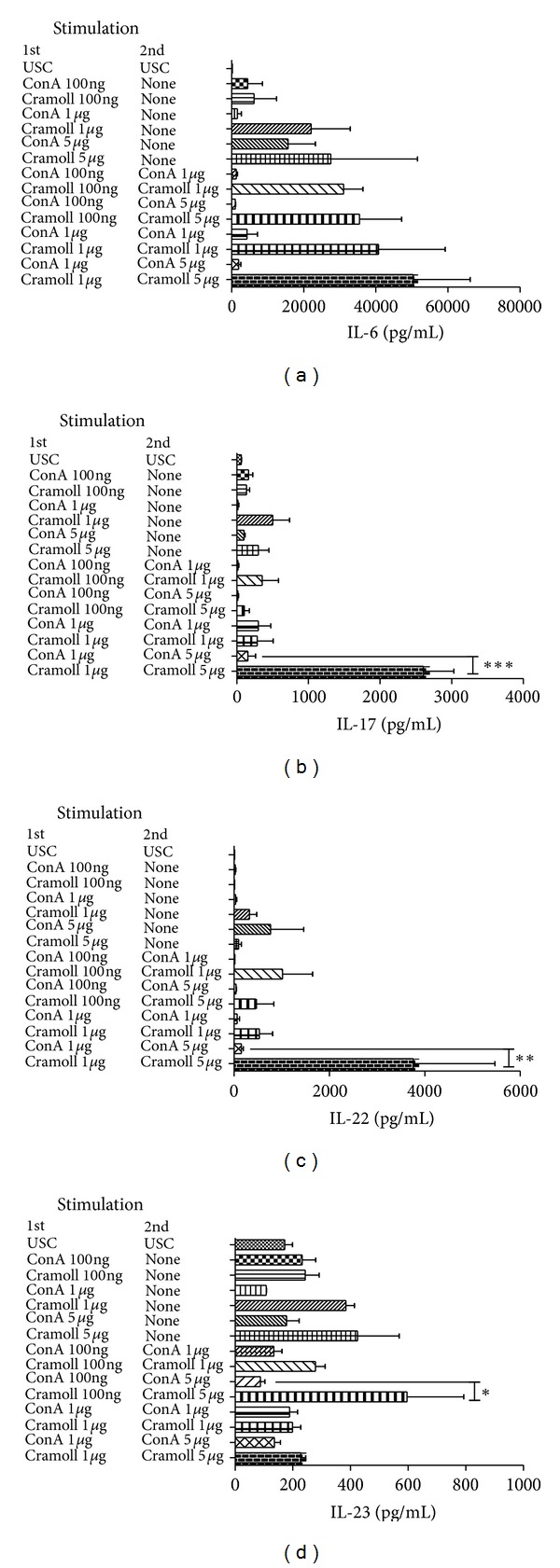
Analysis of immunological memory generation by different concentration of ConA and Cramoll 1, 4 in cultured human PBMC with the second stimulus being provided after five days of the first stimulus and cytokines measured after 24 h.

Assays involving a protocol of previous stimulus followed by a second stimulus can supply information in relation to the immunological memory that the target compound is capable of inducing an *in vitro* memory. Then, after five days of first incubation with different lectin concentrations, the second stimulation was performed during 24 h before cytokines dosage.  Figure 3(a) reveals that when Cramoll 1, 4 and ConA are used as unique stimuli for 6 days (five days + 24 h without second stimulus), Cramoll 1, 4 is more efficient than ConA in stimulating IL-6. The same behavior was obtained when ConA and Cramoll 1, 4 were used as a second stimulus at all concentrations tested; the first and second stimuli with Cramoll l, 4 were the major IL-6 inductor. IL-17 production showed significant differences in the statistical analysis (*P* < 0.001) only when the first stimulus was performed with Cramoll 1, 4 at 1 *μ*g/mL and the second with Cramoll 1, 4 at 5 *μ*g/mL (Figure 3(b)). The same behavior was accompanied by IL-22 that only showed significant differences (*P* < 0.01) at the same condition (Figure 3(c)). The analysis of IL-23 data revealed an interesting result; the best response was obtained when the first stimuli was performed with Cramoll 1, 4 at 100 ng/mL and the second 5 *μ*g/mL (Figure 3(d)) differing from the results obtained by other interleukins analyzed in this study. But like IL-17A and IL-22 there was a significant difference when these IL-23 data were compared with ConA.

## 4. Discussion

Our hypothesis based on IL-6, IL-17A, IL-22, and IL-23 stimulations by lectins is well grounded on the literature that shows many lectins with immunomodulatory effects. The lectin abrin, extracted from *Abrus precatorius* plant, induces murine splenocyte proliferation leading to a Th1 response, activation of NK cells, and stimulation of peritoneal macrophages [15]. Also, the lectin *Abrus* agglutinin (AAG) showed Th1 type immunomodulatory response [16]. Even the stimulatory effect of the Th1 pathway by Cramoll 1, 4 was reported in the literature [5]. These lectin effects have been heavily studied since the 1980s and 1990s [17–19]; however, no work so far observed the potential effect of these molecules towards Th17 pathway.

Initially, we assayed the ability of Cramoll 1, 4 to induce IL-6, IL-17A, and IL-22 secretions in mice splenocytes. IL-6 is a proliferative cytokine, stimulatory of immune cells mainly in the T cells mediated immune response and also, could act as an inductive cytokine of Th17 cell polarization, which in turn produces mainly IL-17 and IL-22 [20, 21]. Cramoll 1, 4 itself has been identified as an immunomodulator in splenocyte cultures from rats treated intraperitoneally with this lectin, increasing ROS production, calcium levels, and expression of IL1B mRNA but not IL-6 or IL-10 [22]. These IL-6 data analyses corroborate with our results. Other work from our group showed that Cramoll 1, 4 at 10 *μ*g/mL revealed higher INF-*γ* production when compared with ConA and revealed an anti-inflammatory response through NO suppression [5]. Recently Abreu and colleagues [23] showed also that in splenocyte cultures 3 different types of algae lectins *Solieria filiformis* (SfL), *Pterocladiella capillacea* (PCL), and *Caulerpa cupressoides* (CCL) induced high levels of IL-6. Stimulation with 1 *μ*g/mL recombinant banana lectin (rBanLec) induced responses by T lymphocyte proliferation and intensive interferon-gamma, interleukin IL-4, and IL-10 secretion in splenocyte culture [24]. So far, this is the first paper in the literature using plant lectins as IL-17 and IL-22 inducers in splenocyte cultures.

Cramoll 1, 4 was effective in stimulating the production of IL-6 better than ConA in human PBMC culture, however no significant difference was observed for IL-17. Concerning IL-22 stimulation Cramoll 1, 4 was more efficient than ConA and in relation to IL-23 showed a high significant difference (*P* < 0.01). This is the first paper to analyze Th17 cytokine path induction in human PBMCs where Cramoll 1, 4 already showed significant mitogenic activity compared with ConA and Phytohemagglutinin (PHA) [2]. Commonly lectins, mainly PHA and ConA, are used in research laboratories as inducing production of cytokines such as IL-1*β*, TNF-*α*, IL-6, IL-2, IFN-*γ*, and GM-CSF in culture of human PBMCs [25–27]. Here we present Cramoll 1, 4 as a biotechnological tool for Th17 related cytokine production and for the first time revealed this ConA property. The mechanism of the immunomodulatory action of Cramoll 1, 4 is not totally known; Cramoll 1, 4 possibly induces the activation of T lymphocytes through transmembrane signals like ConA, since Cramoll 1, 4 has high homology with ConA [28, 29].

Our data showed that the second stimulus with ConA did not induce immunological memory in any assayed conditions for IL-6, IL-17A, IL-22, and IL-23 (Figure 3). Although no statistical difference has been observed between Cramoll 1, 4 and ConA under the conditions studied, it is possible to verify that Cramoll 1, 4 induces a IL-6 dose-dependent response when used as single agent in all tested periods and when used at both time points (Figure 3(a)). These results are in agreement with previous studies from our group in which the first stimulus *in vivo* was carried out intraperitoneally (Cramoll 1, 4 at 100 *μ*L), and the second stimulation performed on splenocytes culture with Cramoll 1, 4 at 10 *μ*g/mL. Then IL-6 was measured 72 h later and six days later [14]. As shown in our results Cramoll 1, 4 was more efficient than ConA in the induction of the response via the IL-6; in both groups this cytokine production was significantly more efficient than that in the unstimulated group.

Cramoll 1, 4 induced significant immunological memory mediating the release of IL-17A and IL-22 when the first stimulus was performed at 5 *μ*g/mL and the second at 1 *μ*g/mL; however only with IL-22 it was possible to observe a dose response. IL-23 results were slightly different than IL-17A and IL-22; the best dose for the first stimuli was 100 ng/mL and for the second was 5 *μ*g/mL. This may be related to the function of maintaining the phenotype Th17 by IL-23 [30] not requiring a large stimulus for pathway maintenance. So we can conclude that Cramoll 1, 4 is able to induce IL-6, IL-17A, IL-22, and IL-23 cytokine production* in vitro* better than ConA, the most widely lectin used nowadays. Furthermore, the results suggested that Cramoll 1, 4 allowed immunologic memory generation by reinducing lymphocyte stimulation.
